# Establishment and Evaluation of a Mouse Model of Experimental Ulcerative Colitis Induced by the Gavage Administration of Dextran Sulfate Sodium

**DOI:** 10.3390/biomedicines12081764

**Published:** 2024-08-05

**Authors:** Dan Wang, Wei Chen, Jie Cao, Luqin Si, Zehong Chen

**Affiliations:** 1 Department of Pharmacy, Union Hospital, Tongji Medical College, Huazhong University of Science and Technology, Wuhan 430022, China; ddw.won@gmail.com (D.W.); chenwei_xh@hust.edu.cn (W.C.); whxh_caojie@163.com (J.C.); 2 Department of Pharmaceutics, School of Pharmacy, Tongji Medical College, Huazhong University of Science and Technology, 13 Hangkong Road, Wuhan 430030, China

**Keywords:** ulcerative colitis, dextran sulfate sodium, gavage administration, dosages, inflammatory indicators

## Abstract

Given the critical role of dextran sulfate sodium (DSS)-induced ulcerative colitis (UC) mouse models in the appraisal of associated therapeutic drugs, the optimization of the administration method and dosages is of paramount importance. Therefore, UC was induced in mice through the gavage administration of a DSS solution instead of free drinking water. The effects of varying daily dosages (2, 4, 6, and 8 g/kg) and frequencies (once or twice) of administration on the body weight and survival rate of the model mice were evaluated. Concurrently, the inflammatory indicators and tissue sections of the model mice were thoroughly evaluated. The results revealed that when the daily dosage reached 8 g/kg, the dosage exhibited a high level of toxicity, resulting in a high mortality rate among the mice. The DSS administration of 6 g/kg*2 not only elicited conspicuous symptoms, significant weight loss, substantial shortening of the colon, and significant changes in various inflammatory indicators, such as myeloperoxidase (MPO), nitric oxide (NO), reactive oxygen species (ROS), and glutathione (GSH), but it also maintained a high survival rate in the UC mice. The findings from this experiment lay a solid experimental foundation for future research on drugs intended for the treatment of UC.

## 1. Introduction

Ulcerative colitis (UC) is classified as a nonspecific chronic inflammatory disease, with the damage primarily confined to the colonic mucosa and submucosa. The primary symptoms include diarrhea, bloody stools, and weight loss [1,2]. The annual incidence rate of UC in Western countries is reported to be as high as 0.5%, and the rate is steadily increasing in emerging industrialized countries, making UC a global health concern [3]. The etiology of UC is multifactorial, with factors such as genetic predisposition, changes in the microbiota, immune system abnormalities, and environmental factors playing significant roles [4,5]. However, the exact pathogenesis remains unclear.

Animal models serve as a crucial tool in modern biomedical research, providing a means to understand the onset and progression of human diseases and to explore preventive and therapeutic measures more effectively. The simulation of the UC model in animal experiments primarily falls into two categories: models of experimentally induced intestinal inflammation and models of spontaneous or genetically engineered intestinal inflammation [6]. An ideal UC model should be economical, convenient, and replicable, exhibiting clinical and histopathological characteristics, treatment responses, and inflammatory mediator characteristics similar to those of human UC. A substantial body of evidence suggests that the etiology, clinical symptoms, pathological changes, and treatment responses of the dextran sulfate sodium (DSS) colitis model closely resemble those of human ulcerative colitis [7]. Therefore, the DSS colitis model has become a critical method for studying the etiology, pathogenesis, and tumor diseases associated with UC and is one of the most widely used UC models currently. The acute or chronic colitis model in mice induced by DSS is typically created using different concentrations of DSS administered through free drinking [8,9,10]. The inherent simplicity and stochastic nature of this modeling approach engender substantial inter-individual disparities among the mice. These disparities subsequently precipitate variations in DSS consumption, thereby culminating in differential manifestations of symptoms in the colitis model mice. This variability can also interfere with the evaluation of the efficacy of therapeutic drugs. This observation underscores the need for a more standardized and controlled experimental design to ensure the reliability and reproducibility of the results [11].

The process of optimizing and investigating the appropriate dosage for the DSS-induced UC mouse model is of paramount importance. It lays a solid groundwork for the evaluation of the therapeutic efficacy of drugs intended for colitis treatment. This study was designed with the aim of enhancing the administration protocol of the DSS-induced UC mouse model, proposing the gavage method as an alternative to the free drinking mode. Our research strategy encompassed the determination of the optimal DSS dosage, the assessment of biochemical markers, and the analysis of tissue sections from the model mice. The ultimate goal was to define the parameters for subsequent studies employing the DSS-induced UC mouse model. This methodology was expected to augment the reproducibility of the DSS-induced colitis mouse model. The knowledge acquired from this study could potentially offer a theoretical framework for the investigation of colitis pathogenesis and drug-based interventions.

## 2. Materials and Methods

### 2.1. Materials and Reagents

DSS was obtained from Sigma Chemical Co. (St. Louis, MO, USA). Polyformaldehyde Radio immunoprecipitation assay (RIPA) lysis buffer and phosphate buffer were purchased from Servicebio company (Wuhan, China). Myeloperoxidase (MPO) assay kit, glutathione (GSH) assay kit, reactive oxygen species (ROS) assay kit, and nitric oxide (NO) assay kit (nitrate reductase method) were purchased from Nanjing Jiancheng Bioengineering Institute (Nanjing, China).

### 2.2. Animals

Male Balb/c mice (23~25 g) were provided by the Experimental Animal Service Center of Sanxia University (Yichang, China). Prior to the commencement of the experiment, the mice were acclimatized for a period of 5 days under a 12 h light/dark cycle. The ambient conditions were meticulously controlled, with the room temperature consistently maintained between 22 and 25 °C and relative humidity kept within the range of 50% to 60%. All experimental procedures involving animals were executed in strict adherence to the guiding principles sanctioned by the Animal Ethics Committee of Huazhong University of Science and Technology, Wuhan (TJ-A2019S956).

### 2.3. Design and Establishment of UC Mouse Model

DSS solutions, freshly prepared with deionized water at varying concentrations (2, 4, 6, and 8 g/kg), were administered to Balb/c mice through oral gavage either once or twice daily over a span of seven consecutive days to induce acute UC [12]. Throughout this period, the mice were allowed unrestricted access to food and water. All mice were randomly divided into 9 groups, with 6 mice in each group. Each group was labeled as X g/kg*N, where X represents the total amount of DSS given to each group of mice per day, and *n* represents the frequency of daily medication, either once or twice a day for DSS administration. The weight of the mice was recorded on a daily basis, and the consistency of their feces, along with the presence of any blood in the stool, was closely monitored. Twenty-four hours subsequent to the final administration, the mice were anesthetized using urethane, and blood samples were collected directly from the heart. The entire blind colon segment was then carefully excised, and its length was accurately measured. The contents of the colon were thoroughly washed with ice-cold PBS, dried using filter paper, and subsequently weighed. A section of the distal colon, approximately 1 cm in thickness, was set aside for H&E staining [13,14]. The remaining tissue was rapidly frozen in liquid nitrogen and preserved at a temperature of −80 °C for future analyses.

### 2.4. Evaluation of Acute UC Mouse Model

#### 2.4.1. Alterations in the Activity and Body Weight of Mice

Systematic observations were carried out on a daily basis to evaluate the luster of the mice’s fur and their psychological condition. In parallel, data related to their weight were diligently documented. Simultaneously, the survival rate of the mice throughout the duration of the experiment was closely monitored to assess the safety of the administered DSS dosage [15]. Weight loss, stool consistency, and rectal bleeding (grade: 0–4) were recorded daily for the disease activity index (DAI) assessment according to Cooper and colleagues [16].

#### 2.4.2. Visual Alterations in the Colon

Twenty-four hours subsequent to the final administration of DSS, images of the excised blind colon segment were obtained, and its length was precisely quantified. Observable alterations in the colonic tissue, including parameters such as color, thickness, and intestinal distension, were meticulously documented [17].

#### 2.4.3. Histopathological and Biochemical Analysis

The freshly extracted distal colonic tissue, approximately 1 cm in thickness, was promptly immersed in 4% paraformaldehyde to facilitate fixation. Following the dehydration of the sample, it was embedded in paraffin and sectioned into slices of 4 μm thickness. These slices were subsequently stained with Hematoxylin and Eosin (H&E) and imaged utilizing a microscope (CX41, Olympus Corporation, Tokyo, Japan) [18,19]. Histological activity index (HAI) scores were evaluated based on the previous literature [20].

#### 2.4.4. Analysis of MPO

The colon tissue was meticulously weighed, and a 5% tissue homogenate was prepared utilizing a specific reagent, strictly adhering to the weight–volume ratio. The reducibility of hydrogen peroxide in MPO was employed to instigate a comprehensive reaction at 37 °C. This was followed by the addition of O-dianisidine to provide hydrogen. The ensuing yellow compound was promptly quantified colorimetrically at 460 nm using a multimode reader (Synergy HT, BioTek Corporation, Broadview, IL, USA), thereby enabling the deduction of MPO activity. It is established that if each gram of wet colon tissue decomposes 1 µmol of H_2_O_2_ in the reaction system maintained at 37 °C, it is considered as one unit of enzyme activity. The formula for calculating the MPO content in the colon is as follows [21]:

(1) $$\mathrm{MPO}\;\mathrm{activity}\;(U/g)=\frac{A_{s}-A_{c}}{11.3\times m}$$

where A_s_ is the absorbance of the measurement group, A_c_ is the absorbance of the control group, and m is the mass of colon tissue.

#### 2.4.5. Analysis of NO

The colon tissue, once sectioned, was subjected to disruption utilizing the RIPA lysis buffer. This was followed by the systematic addition of Griess reagent to the supernatant. Following a period of gentle agitation, the absorbance was quantified at 540 nm. Subsequently, the concentration of NO was computed, with a correction applied for protein concentration, utilizing the following formula [22]:

(2) $$\mathrm{NO}\;\mathrm{level}\;(\mu g/\mathrm{mg})=\frac{\mathrm{NO}\;\mathrm{concentration}\;(\mu g/\mathrm{mL})}{\mathrm{protein}\;\mathrm{concentration}\;(\mathrm{mg}/\mathrm{mL})}$$

#### 2.4.6. Analysis of ROS

The 2′,7′-Dichlorodihydrofluorescein diacetate (DCFH-DA) probe was diluted with PBS and subsequently co-incubated with a homogenate of fresh colon tissue. Upon cellular entry, DCFH-DA underwent hydrolysis by esterase, followed by oxidation by ROS, resulting in the formation of a potent green fluorescent compound. This compound exhibits absorption at an excitation wavelength of 500 nm and an emission wavelength of 530 nm. The ROS content was then calculated, utilizing the following formula [23]:

(3) $$\mathrm{ROS}\;\mathrm{level}=\frac{\mathrm{DCF}\;\mathrm{fluorescence}\;\mathrm{intensity}/\mathrm{mL}}{\mathrm{protein}\;\mathrm{concentration}\;(\mathrm{mg}/\mathrm{mL})}$$

#### 2.4.7. Analysis of GSH

Glutathione, a preeminent thiol compound prevalent in tissues, undergoes a reaction with 5,5′-Dithiobis (2-nitrobenzoic acid) (DTNB) to yield a yellow compound that exhibits a coloration at 420 nm. The concentration of GSH in the tissue, expressed in units per milligram of protein (U/mg protein), was computed based on established standards and subsequently corrected for protein content utilizing the following formula [24]:

(4) $$\mathrm{GSH}\;\mathrm{level}=\frac{\mathrm{GSH}\;\mathrm{concentration}\;(\mu \mathrm{mol}/\mathrm{mL})}{\mathrm{protein}\;\mathrm{concentration}\;(\mathrm{mg}/\mathrm{mL})}$$

### 2.5. Statistical Analysis

All experiments were performed in at least three independent evaluations, and the data are displayed as the mean ± SEM. Significant differences were determined by the *t*-test, and *p* < 0.05 was considered significant.

## 3. Results

### 3.1. Mouse Weight and Survival Rate

**Figure 1:**
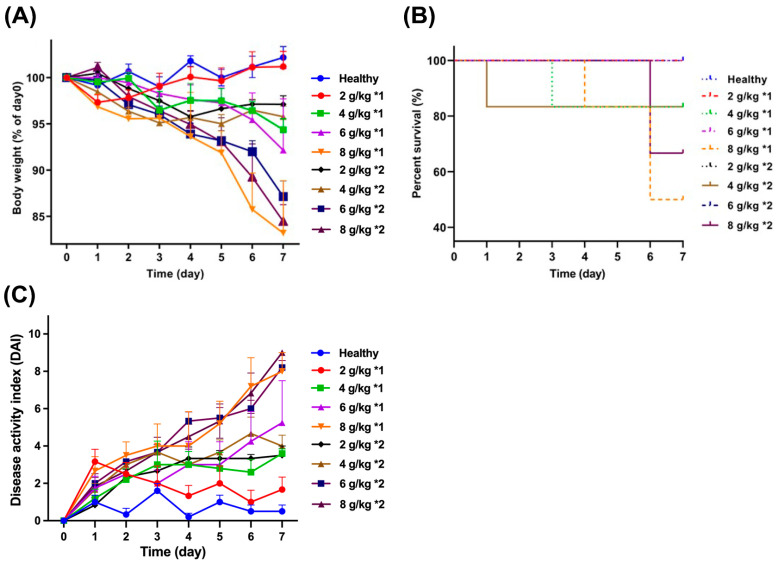
Time-dependent variations of body weight (**A**), percent survival rate (**B**), and DAI value (**C**) of mice treated with DSS at different dosages and frequencies.

UC was induced in mice through the gavage administration of daily dosages (2, 4, 6, and 8 g/kg) of DSS, with the objective of investigating the impact of different dosing frequencies (administered either once or twice daily) on the weight and survival rate of the mice shown in Figure 1. After 7 days of treatment, it was observed that the mice treated with 8 g/kg*1 and 8 g/kg*2 experienced the most significant weight loss, with their weights reducing to 83.2% and 84.5% of the weight of healthy mice, respectively. Concurrently, the mortality rate among the DSS mice that received a dosage of 8 g/kg, administered either once or twice daily, was notably high, standing at 66.7% and 50.0%, respectively. The results indicated that the aforementioned dosages exhibit a high level of toxicity, resulting in a high mortality rate among the mice. In contrast, a daily dosage of 2 g/kg did not result in any significant changes in the weight and survival rate of the mice when compared to healthy mice. Furthermore, while mortality was also observed among mice that received daily dosages of 4 and 6 g/kg, the overall survival rate was comparatively higher. The DAI value of the mouse model treated with DSS at different dosages and frequencies is shown in Figure 1C. The results indicated that the DAI value of model mice was higher than that of the healthy group due to the inflammation introduced by DSS. Notably, a significant increase in the DAI value was observed when the daily dosage reached 6 g/kg (Table 1).

### 3.2. Length of Mouse Colon

**Figure 2:**
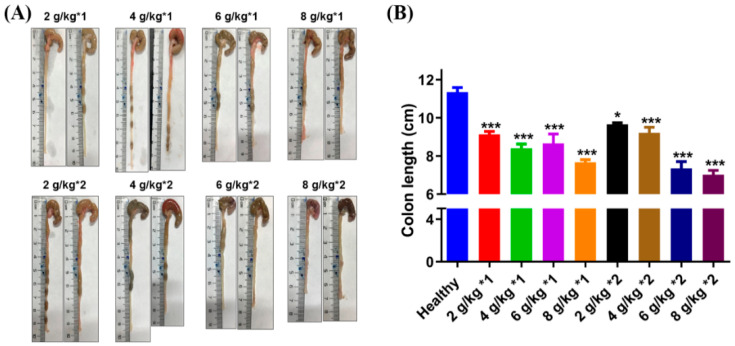
Colon images (**A**) and colon length (**B**) of mice treated with different daily dosages and frequencies of DSS. * *p* < 0.05 and *** *p* < 0.001 vs. healthy group.

As shown in Figure 2, the colon length of the healthy group was measured to be 11.35 ± 0.6 cm. A decrease in colon length was observed across all treatment groups to varying extents, as detailed in Table 1. Under the premise of a satisfactory survival rate in the model mice, a significant reduction in colon length to 7.67 ± 0.25 cm was noted when the daily dose was administered as 6 g/kg in two separate doses. Concurrently, the colon images revealed noticeable swelling in the cecum of mice treated with DSS. Upon increasing the daily dose to 6 or 8 g/kg, the mice exhibited a substantial shortening and thinning of the colon, reduced intestinal contents, and a propensity for congestion in the distal colon, as shown in Figure 2A.

### 3.3. Colon Tissue Section

**Figure 3:**
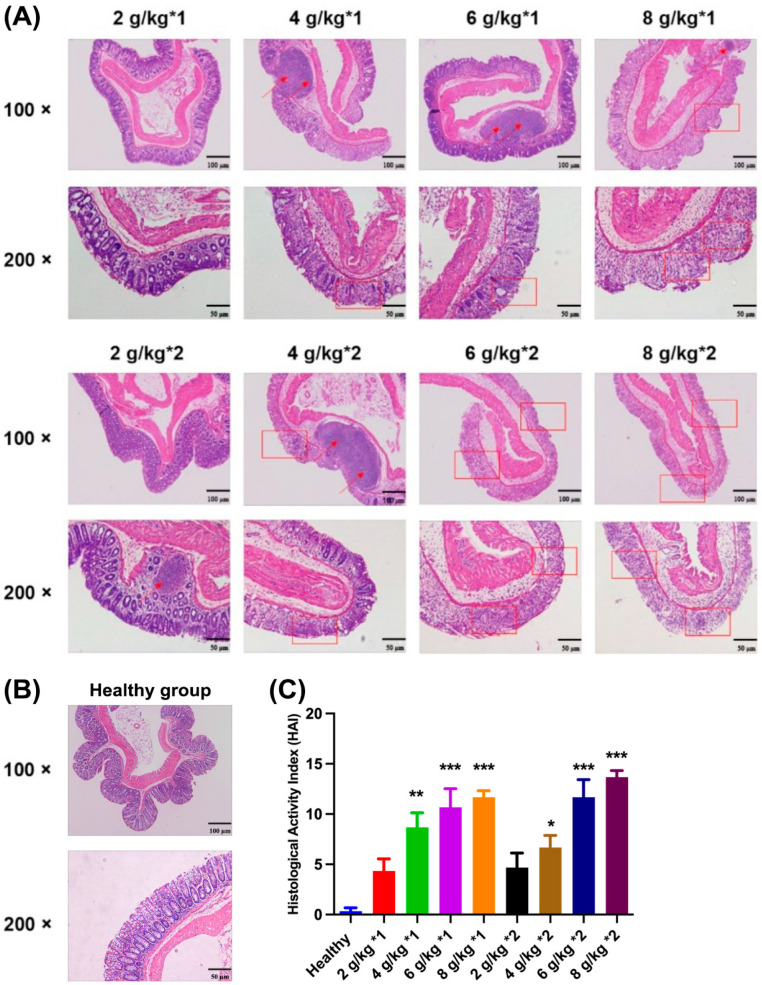
Colon tissue section of mice treated with different daily dosages and frequencies of DSS (**A**) and healthy mice (**B**). (The arrow indicates inflammatory infiltration while the rectangle indicates a disorder of the crypt structure). The HAI scores of colon sections (**C**). * *p* < 0.05, ** *p* < 0.01, and *** *p* < 0.001 vs. healthy group.

Upon microscopic examination of the colon tissue, the physiological structure of the normal colon tissue appeared to be intact and distinct. The goblet cells were systematically arranged within the mucosal tissue, and the crypt structure was essentially normal, as detailed in Figure 3B. However, with an escalating daily dosage of DSS, a gradual dissolution of goblet cells within the colon tissue structure, a disappearance of crypt structures, and, in some instances, an infiltration of inflammatory cells were observed (Figure 3A). When the dosage reached 8 g/kg, the colon epithelium began to exhibit irregularities, accompanied by the partial destruction and shedding of the epithelial tissue. Similarly, the colon HAI scores (Figure 3C) further confirmed the trend of positive correlation between DSS-induced intestinal inflammation and daily dose. Specifically, when the daily dosage reached 4 g/kg, the HAI score significantly increased compared with the healthy group.

### 3.4. Inflammatory Indicators of UC

**Figure 4:**
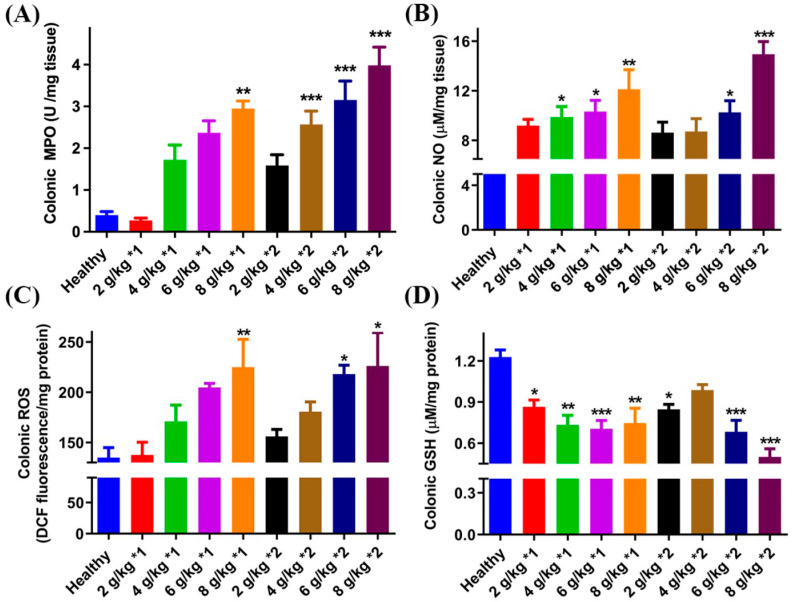
Colon inflammatory indicators MPO (**A**), NO (**B**), ROS (**C**), and GSH (**D**) levels of mice treated with different daily dosages and frequencies of DSS. * *p* < 0.05, ** *p* < 0.01, and *** *p* < 0.01 vs. healthy group.

The results of the inflammatory indicators for the colitis mice are shown in Figure 4. The MPO levels exhibited a positive correlation with the DSS dose. Furthermore, administering the same daily dose of DSS in two divided doses, rather than once a day, led to increased inflammation levels in mice. Specifically, when the frequency of administration was twice daily and the daily dosage reached 4 g/kg, the MPO level in the model mice significantly exceeded that of the healthy group. The trends in NO and ROS levels in the colon were analogous, with substantial differences only noticeable when the DSS modeling dose reached 6 g/kg and 8 g/kg. No significant distinction was observed between once-daily and twice-daily administration. Aside from the group treated twice daily at 4 g/kg, all other groups exhibited significant changes in colon GSH content (*p* < 0.001 for 6 g/kg*1, 6 g/kg*2, and 8 g/kg*2).

In light of the aforementioned indicators and various factors, including the survival rate of the mice, it was ascertained that the administration of a daily dosage of 6 g/kg, divided into two doses, could effectively induce a mouse model of colitis while maintaining a relatively high survival rate. These research findings served as the foundation for a subsequent study, which successfully induced a mouse model of colitis using the same dosage regimen [25]. This subsequent study further explored the biological activity of emodin nanoparticles in the treatment of colitis, thereby expanding our understanding of potential therapeutic strategies.

## 4. Discussion

The establishment and optimization of UC models are crucial for exploring drug efficacy and potential mechanisms. However, the task of identifying an appropriate UC mouse model for preclinical research is not straightforward. Various UC mouse models are in use, including those based on chemical induction, spontaneous mutation, adoptive T cell transfer, genetic engineering, and microbial induction [26,27]. Each modeling method can replicate different characteristics of inflammatory bowel disease (IBD) to varying degrees, considering factors such as histology, efficacy, and disease location [7]. Chemically induced disease models, such as those using DSS, Trinitrobenzene sulfonic acid (TNBS), and Oxazolone, are frequently employed in preclinical mouse models of UC. TNBS triggers Th1/Th17 cell responses through transmural inflammation, leading to severe colitis symptoms. Oxazolone can induce IL-13-mediated Th2 responses, and both are commonly used to investigate CD4^+^ T cell-dependent immune responses [28]. The DSS-induced model, due to its ease of operation, repeatability, and resemblance to the pathological characteristics of human UC, has become a classic model in UC research. It is characterized by ulcers and granulocyte infiltration and is often used to study the mechanisms of acute and chronic colitis development, as well as drug efficacy. Zhang et al. found that Bilobalide, a component of Ginkgo extract, could alleviate the inflammatory response in UC mice introduced by DSS, enhance the repair of intestinal epithelial barrier function, and reshape the intestinal microbial community, thereby mitigating the progression of UC [29].

The assessment of UC severity is commonly based on two key indicators: weight fluctuation and survival rate. The induction of colitis in mice using DSS often involves administering an excessive dose. Herein, a daily dose of 8 g/kg can induce extreme inflammation in mice, resulting in irreversible damage and hindering the accurate evaluation of therapeutic drug efficacy. On the other hand, an insufficient dosage, such as a daily dosage of 2 g/kg, may result in less noticeable colitis symptoms in mice, thereby compromising the effectiveness and scientific basis of the treatment drugs. A comparative study was conducted on a mouse model of colitis induced by DSS solutions of 1.5%, 2.5%, and 3.5% (*w*/*v*). The study revealed that a 3.5% DSS solution can cause severe epithelial tissue damage, leading to mouse mortality and potentially irreversible chronic inflammation [30]. However, another study successfully induced a colitis mouse model using a 3.5% DSS solution [31]. Compared to healthy mice on a standard diet, the body weight of these mice did not significantly decrease. As reported in the literature, modulating the concentration and frequency of DSS administration can lead to varying degrees of colon inflammation [32]. Our findings demonstrated that increasing the administration frequency, such as administering a daily dose of 8 mg/kg twice, effectively mitigates the toxicity associated with high DSS doses, resulting in an improved mouse survival rate (from 33.3% to 50%). Meanwhile, maintaining a consistent daily dose of DSS sustains the induced colon inflammation, leading to comparable weight loss levels in mice. Additionally, it is clear that the free drinking water method results in the poor reproducibility of experimental results, and also increases the coefficient of variation between mouse groups. Moreover, due to individual differences, free drinking water may lead to variations in the doses of DSS ingested by different mice, thus resulting in different colitis courses. Shimizu et al. suggested that varying doses of free drinking water, such as a DSS content of 2%, 3%, and 4%, may lead to mild, moderate, and severe UC disease courses in weaned rats, respectively [33]. When administering the DSS solution via gavage, groups were formed based on the dose per unit body weight. This approach ensures that mice in the same group receive a similar amount of the inducer DSS, providing a more scientific method for evaluating drug effectiveness.

In colitis mouse models, it has been observed that the length of the mouse colon is significantly reduced due to inflammation [34]. This is accompanied by symptoms such as bleeding and edema in the colon tissue. In the state of disease, the mice exhibit compromised intestinal function, leading to symptoms like loose stools and bloody stools. The study revealed that when the daily dosage reached 8 g/kg, the intestinal function of the mice was severely impaired, and the length of the mouse colon was drastically shortened. This irreversible damage also increased the mortality rate among the mice. Therefore, it is suggested that a daily dosage of less than 6 g/kg can induce a colitis mouse model while ensuring a reasonable survival rate of the mice. Inflammation can lead to damage to colonic mucosal cells, including the loss of goblet cells and the loss of crypts [35,36]. The damage and loss of these cells can disrupt the normal functions of the colon, including food digestion and absorption, and water absorption. The length of the colon can be reduced due to inflammation and cell damage. Moreover, inflammation can cause an imbalance in the gut microbiota, which may also affect the length of the colon [37]. The gut microbiota plays a crucial role in maintaining intestinal health and function, including maintaining the integrity of the intestinal mucosa, inhibiting the growth of harmful bacteria, and participating in the metabolism of nutrients. Therefore, the dysbiosis of the gut microbiota may further exacerbate the inflammatory response of the colon, resulting in a shortening of the colon length.

Inflammatory markers are pivotal in evaluating UC models. A key feature of UC is the accumulation of neutrophils in the inflamed intestinal mucosa. MPO serves as both a functional and an activation marker for neutrophils [38]. NO, a novel immune molecule and inflammatory transmitter, can mediate inflammation and tissue damage within the body [39]. In tissues affected by UC, there is often a significant increase in the concentration of ROS, while the level of the antioxidant GSH is notably reduced. Considering all these indicators, modeling with DSS at a dosage of 6 g/kg*2 can enhance the visibility of UC symptoms, reduce animal mortality, and conserve the dosage of modeling agents. Therefore, it presents a reliable method for modeling in subsequent research.

## 5. Conclusions

This study utilized the gavage administration of DSS to establish a mouse model of UC. The effects of different daily dosages and frequencies of administration were scrutinized. The application of 6 g/kg*2 of DSS for modeling UC led to the appearance of distinct symptoms in the mice, along with substantial alterations in various inflammatory indicators. Simultaneously, this method allowed for a reduction in animal mortality and the conservation of the dosage of modeling agents. The results of this experiment lay a solid experimental groundwork for subsequent research into therapeutics for UC.

## Figures and Tables

**Table 1:** Physical characteristics of mice with different daily dosages and frequencies of DSS.

Dosage (g/kg)	Frequency	Body Weight Percentage	Survival Rate	DAI	Colon Length (cm)
2	1	101.2 ± 1.65	100	1.67 ± 0.67	9.13 ± 0.16 ***
	2	97.1 ± 0.93	100	3.50 ± 0.50	9.66 ± 0.09 *
4	1	94.4 ± 1.21 **	83.3	3.60 ± 0.24	8.40 ± 0.23 ***
	2	95.8 ± 1.14	83.3	4.00 ± 0.58	9.22 ± 0.29 ***
6	1	92.2 ± 5.55 ***	100	5.25 ± 2.25	8.67 ± 0.49 ***
	2	87.1 ± 1.69 ***	83.3	8.20 ± 0.37 **	7.35 ± 0.36 ***
8	1	83.2 ± 5.66 ***	33.3	8.00 ± 1.00 **	7.67 ± 0.15 ***
	2	84.5 ± 1.18 ***	50.0	9.00 ± 0.00 **	7.03 ± 0.23 ***

Note: * *p* < 0.05, ** *p* < 0.01, and *** *p* < 0.001 vs. healthy group.

## Data Availability

The original contributions presented in the study are included in the article, and further inquiries can be directed to the corresponding authors.
